# Efficacy of different doses of oliceridine for Patient-Controlled Intravenous Analgesia in patients undergoing Total Laparoscopic Hysterectomy

**DOI:** 10.1371/journal.pone.0357372

**Published:** 2026-09-03

**Authors:** Jiaojiao Deng, Baobao Ma, Kaihua Wei, Tao Wei, Liping Xie, Hong Zhang, Changxu Wang

**Affiliations:** 1 Department of Anesthesiology, First Affiliated Hospital of Shihezi University School of Medicine, Shihezi, China; 2 Department of Anesthesiology, The Cancer Hospital Affiliated to Chongqing University, Chongqing, China; Public Library of Science, UNITED STATES OF AMERICA

## Abstract

**Objective:**

To evaluate the efficacy and safety of three oliceridine different doses for oliceridine for patient-controlled analgesia (PCIA) in patients undergoing during total laparoscopic hysterectomy, following the premature termination of the planned sufentanil control arm.

**Methods:**

This prospective, randomized, double-blind trial was originally designed as a four-arm study. Due to operational challenges in patient recruitment and randomization within the single-center setting-specifically, significant reluctance among eligible patients to be randomized to receive a traditional opioid regimen when oliceridine was available, leading to unsustainable enrollment timelines—the sufentanil control arm was prematurely terminated. The final analysis therefore focused on three oliceridine doses: 0.1, 0.35, and 0.5 mg/kg, diluted to 100 ml with normal saline for 48-hour PCIA. The primary endpoint for sample size calculation was the incidence of postoperative nausea and vomiting (PONV) at 24 h. Efficacy and safety outcomes analyzed included: NRS pain scores (at rest and during cough), Ramsay sedation scores, incidence of rescue analgesia, various recovery indicators (time to first flatus, time to ambulation, QoR-15 scores), and opioid-related adverse events.

**Results:**

Compared to the low-dose group, the moderate- and high-dose groups demonstrated significant improvements in cough-pain scores and bolus press attempts (“Bolus press attempts” were defined as the total number of patient-initiated demands for a supplemental dose, including attempts made within the lockout interval) (*p* < 0.0083). Further comparison revealed that the moderate-dose group showed the best recovery outcomes, including shorter time to first flatus and ambulation, and higher QoR-15 scores. In contrast, although the high-dose group provided more potent analgesia, it was associated with a higher incidence of nausea, delayed flatus, and a significantly lower quality of recovery (QoR-15) score at 48 hours postoperatively compared to the moderate-dose group (*p* < 0.0083).

**Conclusion:**

Within the investigated PCA oliceridine dose range, the 0.35 mg/kg dose promotes early recovery in patients undergoing laparoscopic total hysterectomy through effective analgesia and reduced nausea, representing the best balance between efficacy and safety among the tested oliceridine-specific regimens.

## Introduction

Approximately 86% of patients experience postoperative pain, with 75% reporting moderate to severe pain immediately after surgery. In addition, more than 80% of patients fail to achieve adequate pain control postoperatively [1,2]. This not only impacts quality of life and functional recovery but may also lead to delayed recovery, prolonged hospital stays, and increased healthcare costs [3–5]. With the widespread adoption of laparoscopic surgery, despite its reduced invasiveness, early postoperative pain may be comparable to or even more severe than that following open surgery. Therefore, preventing and managing laparoscopic postoperative pain is particularly crucial [6,7]. Patient-controlled intravenous analgesia (PCIA) has become a common method for managing moderate to severe postoperative pain using opioids. It is easy to operate and allows on-demand dose adjustment to maintain effective blood concentrations, providing continuous analgesic effects [8,9]. However, the utility of PCIA is often limited by dose-dependent adverse reactions such as respiratory depression, nausea, vomiting, and gastrointestinal paralysis [10].

Currently used opioid agonists (e.g., morphine and fentanyl) simultaneously activate both G protein– and β-arrestin–mediated signaling pathways. In contrast, oliceridine, a G protein–biased agonist of the μ-opioid receptor (MOP), preferentially engages G protein signaling and exhibits markedly reduced β-arrestin recruitment (particularly β-arrestin-2) compared with morphine [11]. Theoretically, this biased signaling profile may afford robust analgesia while reducing adverse events associated with β-arrestin-2 pathway activation, such as nausea/vomiting and respiratory depression [12,13]. Studies have confirmed oliceridine’s efficacy in treating moderate-to-severe acute pain, as well as its potential to decrease the incidence of nausea/vomiting and mitigate the risk of respiratory depression [14–17]. However, its application in gynecologic populations—particularly in laparoscopic surgeries, which are associated with high rates of postoperative nausea and vomiting [18]—remains understudied.

Currently, research on oliceridine for postoperative analgesia has primarily focused on bolus dosing without a background infusion. The recommended single-bolus dose is 0.35 mg with a 6-minute lockout period, and the daily cumulative dose should not exceed 27 mg [19]. Patient gender, age, and body weight have no significant impact on oliceridine plasma concentrations, and there is currently no evidence supporting weight-based dosing regimens [20,21]. Despite its promising pharmacologic profile, the optimal dose of oliceridine for PCIA in laparoscopic gynecological populations—particularly in procedures associated with a high baseline risk of postoperative nausea and vomiting (PONV), such as total laparoscopic hysterectomy (TLH)—remains undefined. While pivotal trials (e.g., APOLLO, ATHENA) have established its efficacy in abdominal wall and orthopedic surgeries, a systematic evaluation of its dose-response relationship and gastrointestinal tolerability within the context of ERAS-focused gynecologic laparoscopy is lacking. This absence of procedure-specific data limits the evidence-based integration of oliceridine into enhanced recovery protocols for gynecological patients.

To address gaps in the existing evidence, we conducted a randomized controlled trial that was originally designed as a four-arm study (including a sufentanil control arm). Due to unforeseen challenges in recruiting patients willing to be randomized to a standard opioid regimen, the sufentanil arm was discontinued. This analysis focuses on systematically evaluating the efficacy and safety of three different doses of oliceridine (0.1, 0.35, and 0.5 mg/kg) for PCIA following laparoscopic total hysterectomy. This study aims to explore the safest and most effective regimen among these oliceridine-specific PCIA regimens for patients undergoing laparoscopic total hysterectomy and to provide high-quality clinical evidence to support the implementation of Enhanced Recovery After Surgery (ERAS) protocols in gynecologic laparoscopic procedures.

## Methods and materials

### Study design and ethics

This single-center, prospective, randomized, double-blind clinical trial was originally designed as a four-arm study (including a sufentanil control arm). Ethical approval for this study was granted by The Scientific and Technology Ethics Committee of The First Affiliated Hospital of Shihezi University (Medical Ethics Approval No.: KJ-2024-514-02). All subjects provided written informed consent prior to any study-related procedures. This manuscript adheres to the CONSORT (Consolidated Guidelines for Reporting Trials) guidelines. The recruitment period for this study was from 20/02/2025 to 31/08/2025.

### Trial registration

The trial protocol was prospectively registered with the China Clinical Trial Registry prior to subject enrollment (Registration Number: ChiCTR2500097470; Principal Investigator: Jiaojiao Deng; Registration Date: February 19, 2025). Although the initial registration was completed retrospectively due to administrative delays in the finalization of documents during the institutional approval process, the authors confirm that all ongoing and related trials for this drug/intervention are registered. Due to unforeseen challenges in recruiting patients willing to be randomized to a standard opioid regimen, the sufentanil arm was prematurely terminated. Consequently, the final analysis focused on three oliceridine doses. However, the statistical analysis framework adheres to the original four-arm design (assuming six pairwise comparisons) to maintain methodological rigor.

### Participants

This prospective trial enrolled patients with American Society of Anesthesiologists physical status I-II, aged 18 ~ 65 years, body mass index (BMI) 18.5 ~ 35 kg/m², and body weight >40 kg. Participants underwent elective laparoscopic hysterectomy. The study was approved by the Institutional Review Board (IRB). as a four-arm trial; however, data analysis focused on the three oliceridine dose groups due to the discontinuation of the sufentanil arm. All participants provided written informed consent. All participants provided written informed consent. Exclusion criteria included: severe cardiopulmonary, hepatic, or renal dysfunction (New York Heart Association functional class ≥ III, chronic obstructive pulmonary disease, Child-Pugh grade ≥ B, estimated glomerular filtration rate < 30 mL·min^-1^·1.73 m^-^²), neuropsychiatric comorbidities (dementia, schizophrenia, long-term use of psychotropic analgesics >3 months), communication-impairing neurological deficits (post-stroke sequelae, aphasia), preoperative hypoxemia (PaO_2_ < 60 mmHg, SpO_2_ < 92% on room air), recent participation in other drug trials (≤30 days), and known or suspected gastrointestinal obstruction. Protocol deviations leading to discontinuation included: surgery duration exceeding 3 hours, hypersensitivity reactions, blood loss >800 mL, unplanned reoperation, non-compliance with the analgesia protocol, or failure to complete the 48-hour postoperative assessment.

### Randomization and blinding

A random number table method was employed, assigning patients to one of four groups (three oliceridine doses and one sufentanil control) based on their enrollment sequence. Due to the premature termination of the sufentanil arm, the final analysis focused on the three oliceridine doses. An independent statistician outside the research team generated the allocation sequence and sealed the results in sequentially numbered, opaque envelopes. On the day of surgery, a blinded medication administrator—uninvolved in any study procedures—opened the envelopes and provided the corresponding numbered study medication (pre-prepared by the pharmacy per the randomized protocol, with identical appearance) to the medication preparer. The medication preparer, who only verified the patient ID and remained unaware of the group assignment, prepared the drug and delivered it to the anesthesiologist. The drug was labeled “Study Drug” with the patient ID. Throughout the process, the patient, anesthesiologist, surgeon, postoperative follow-up personnel, and data collectors remained unaware of the group assignment.

### Preoperative management

Another anesthesiologist, unaware of the randomized sequence, provided standardized training on the NRS (0 = no pain to 10 = worst pain) pain intensity scoring tool during the preoperative visit for patients enrolled in this trial. The anesthesiologist informed the patients and their families about the planned anesthesia method, explained the trial procedures, safety, risks, and precautions, and obtained their understanding and cooperation. After securing informed consent from the patients and their families, the anesthesiologist guided them to sign the informed consent form.

### Anesthesia method

Patients underwent routine preoperative fasting for 8 hours and fluid restriction for 4 hours. Upon entering the operating room, standard cardiac monitoring was initiated, with continuous monitoring of blood pressure, heart rate, and blood oxygen saturation. After preparing emergency medications and equipment, general anesthesia induction was performed. Following mask oxygenation with denitrogenation, the following agents were administered intravenously sequentially: midazolam 0.05 mg/kg, etomidate 0.2 mg/kg, sufentanil citrate 0.5 μg/kg, and rocuronium bromide 0.6 mg/kg. Endotracheal intubation was performed once the patient lost consciousness, exhibited absent corneal reflexes, and demonstrated jaw relaxation. Following successful intubation, mechanical ventilation was initiated with respiratory parameters adjusted based on PETCO_2_ to maintain a target range of 35 ~ 45 mmHg. Target-controlled infusion of propofol (4 ~ 6 mg/kg/h) and remifentanil (0.15 ~ 0.30 μg/kg/min) was used to control anesthesia depth, maintaining the Bispectral Index (BIS) between 40 and 60. Concurrently, neuromuscular blockade was maintained with continuous infusion of cisatracurium besylate at 1 ~ 2 μg/kg/min as required for surgical needs. Throughout the procedure, blood pressure was maintained within 20% of baseline measurements based on BIS values and by adjusting intravenous infusion rates. Intravenous vasoactive agents were initiated immediately if any hemodynamic variable deviated more than 20% from baseline measurements. Hypotension [defined as systolic blood pressure (SBP) <90 mmHg or >20% below baseline] was treated with 40 μg phenylephrine [heart rate (HR) >60 bpm] or 6 mg ephedrine (HR ≤ 60 bpm). While hypertension (SBP > 160 mmHg or >20% above baseline) was treated with 0.1 mg/kg diltiazem. Bradycardia (HR < 50 bpm) was corrected with intravenous atropine 0.3 ~ 0.5 mg.

Anesthetics were discontinued during skin closure. Following surgery, patients were transferred to the post-anesthesia care unit (PACU). Extubation criteria included restoration of muscle strength and consciousness, clearance of airway secretions, and manual lung expansion. Although the study was originally designed as a four-arm trial, the sufentanil arm was discontinued. After extubation, all three groups received a loading dose of 1.5 mg oliceridine intravenously, consistent with dosing strategies employed in prior pivotal trials to ensure rapid attainment of therapeutic plasma levels before PCIA initiation. Oxygen administration ceased after 5 minutes, and postoperative analgesia was initiated.

Patients in the group L received PCIA: 0.1 mg/kg oliceridine dissolved in 100 mL saline (baseline rate 2 mL/h, bolus 0.5 mL, lockout 15 min); Group M patients received PCIA: 0.35 mg/kg oliceridine dissolved in 100 mL saline (baseline rate 2 mL/h, bolus 0.5 mL, lockout 15 min); Group H patients received PCIA: 0.5 mg/kg oliceridine dissolved in 100 mL saline (baseline rate 2 mL/h, bolus 0.5 mL, lockout 15 min). Monitoring continued in the PACU until the Steward recovery score exceeded 4. Patients were ultimately transferred back to the ward. When postoperative NRS pain scores reached ≥4, intravenous tramadol (1 mg/kg) was administered as a rescue analgesic.

### Outcome measures

The primary endpoint was the incidence of postoperative nausea and vomiting (PONV) at 24 hours, consistent with the four-arm protocol design used for sample size calculation. Secondary outcomes were categorized into three domains:

Pain and Sedation: NRS pain scores (at rest and during cough), Ramsay sedation scores at 0.5, 6, 12, 24, and 48 hours post-loading dose, and bolus press attempts —defined as effective presses (resulting in drug delivery) and total presses (all demands regardless of lockout)—were analyzed.

Recovery Indicators: Postoperative recovery quality assessed at 24 and 48 hours using the validated 15-item Quality of Recovery (QoR-15) scale (total score range 0 ~ 150; higher scores indicate better postoperative recovery). Time to first flatus and ambulation were also recorded.

Safety: Adverse events within 48 hours, including nausea, vomiting, respiratory depression, dizziness and pruritus. Constipation was defined as failure to defecate within 48 hours postoperatively.

Exploratory Variables: Time to regain consciousness and hemodynamic parameters (HR, MAP, SBP) were recorded as standard anesthesia monitoring data. This structure ensures all reported outcomes align with study’s dual objectives: evaluating the dose-response relationship of oliceridine for PCIA in laparoscopic hysterectomy and identifying the optimal dose balancing analgesia and ORAEs, based on the original four-group analytical framework.

### Sample size calculation

Sample size was calculated using PASS 15 software. Based on a literature review [22], the anticipated incidence of postoperative nausea and vomiting (PONV) at 24 h was 20% (0.1 mg), 30% (0.35 mg), and 42% (0.5 mg). The primary endpoint was the 24 h PONV incidence. With α = 0.05 and 1-β = 0.8 (power = 80%), strict adherence to the Four-Group Framework: Although the final analysis focused on three oliceridine groups, the statistical analysis framework adheres to the original four-arm design to maintain methodological rigor. Accounting for a 20% dropout rate, the target enrollment was 43 patients per group. Although the study was originally designed as a four-arm trial, the final sample size of 42 patients per oliceridine group nearly met this target (43) and exceeds the minimum requirement of 36 patients per group, ensuring adequate statistical power for the dose-comparison analysis.

### Statistical analysis

Continuous variables were tested for normality using the Shapiro-Wilk test. Normally distributed data are presented as mean ± standard deviation (mean ± SD) and compared using one-way analysis of variance (ANOVA), followed by Bonferroni post-hoc tests for pairwise comparisons (assuming six comparisons in accordance with the original four-group framework). Non-normally distributed data were expressed as median and interquartile range [M (Q1, Q3)] and compared using the Kruskal-Wallis test, with pairwise comparisons using the Bonferroni-corrected Wilcoxon rank-sum test (assuming six comparisons).

Categorical variables were expressed as cases (%) and compared between groups using the χ^2^ test or Fisher’s exact test.

Note: Although the study protocol was designed for four arms, the final analysis focused on three oliceridine groups. The statistical framework adheres to the original four-arm design by applying Bonferroni correction for the six pairwise comparisons (adjusted α = 0.0083). Adjusted *P* < 0.0083 was considered statistically significant.

## Results

### Study population

**Fig 1:**
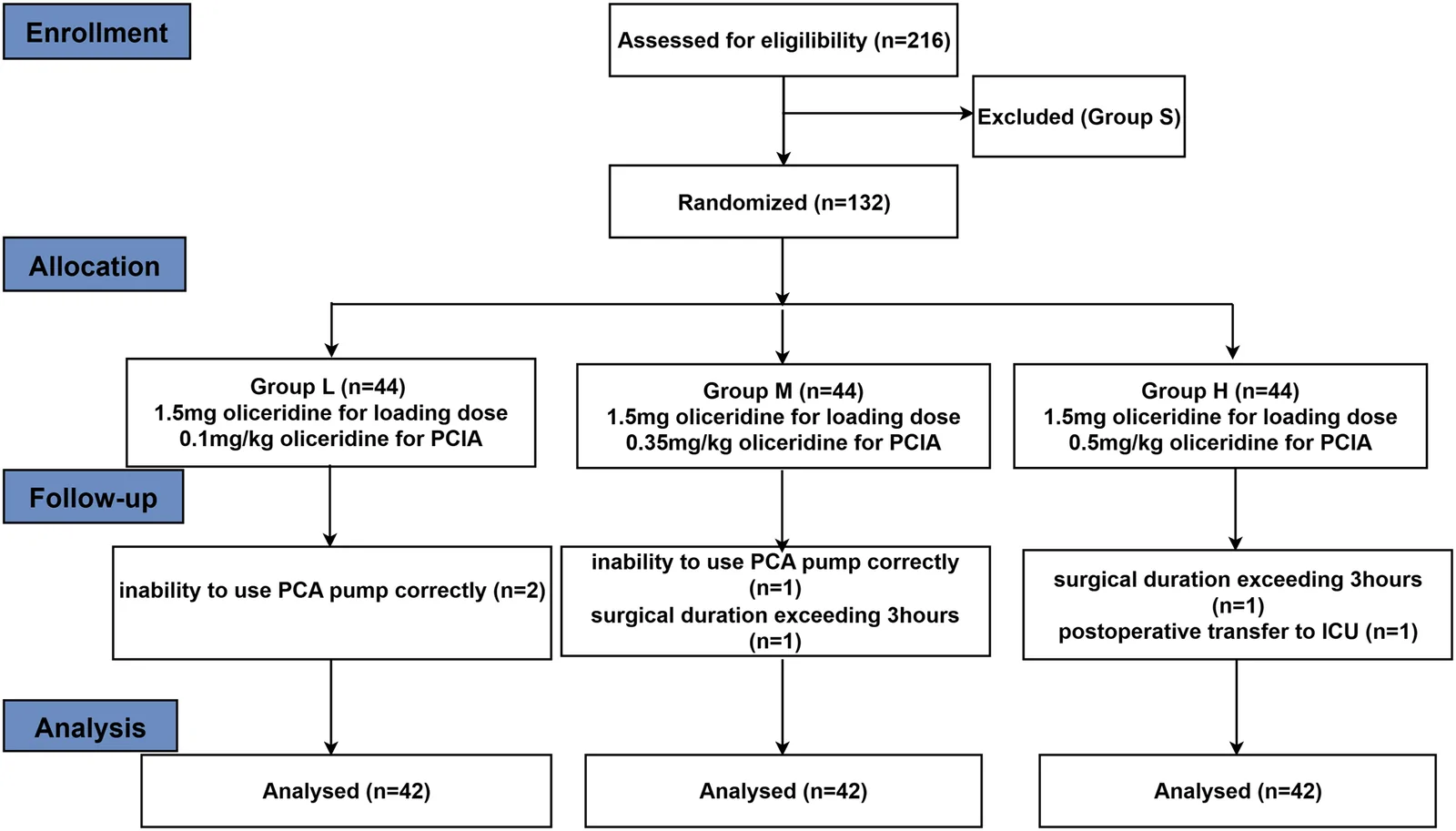
The flow chart of the study.

The study was originally designed to recruit 54 patients per group for four arms. Due to premature termination of the sufentanil arm, data analysis focused on the three oliceridine doses. Exclusions after randomization were as follows: In the L group, 2 patients failed to use the analgesic pump correctly; in the M group, 1 patient used the analgesic pump incorrectly and 1 patient had a surgical duration exceeding 3 hours; in the H group, 1 patient had a surgical duration exceeding 3 hours and 1 patient was transferred to the ICU postoperatively. Consequently, the final sample size comprised 126 patients (42 per oliceridine group). Although the target enrollment was 54 patients per group (accounting for a 20% dropout rate based on the original four-arm protocol), (based on 20% dropout) and exceeded the minimum requirement of 36 patients per group (calculated for the three-group framework without dropout), ensuring adequate statistical power for the dose-comparison analysis (Fig 1).

No statistically significant differences were observed among the three groups in terms of age, height, weight, BMI, history of motion sickness, history of abdominal surgery, Apfel score, duration of pneumoperitoneum, surgical duration, blood loss, or length of hospital stay (Table 1).

**Table 1:** Baseline data.

Variables	Group L (n = 42)	Group M (n = 42)	Group H (n = 42)	F/χ^2^	*P*
Age, yr	51.60 ± 5.50	52.79 ± 5.54	50.57 ± 7.25	0.89	>0.99
Height, m	1.56 ± 0.06	1.59 ± 0.05	1.59 ± 0.05	2.45	>0.99
Weight, kg	63.19 ± 5.33	63.67 ± 6.64	63.12 ± 5.70	0.02	>0.99
BMI, kg/㎡	24.99 ± 1.91	25.25 ± 2.49	24.98 ± 2.25	0.45	>0.99
Apfel	3.38 ± 0.49	3.38 ± 0.49	3.14 ± 0.52	1.85	>0.99
Operation duration, min	109.79 ± 29.91	110.52 ± 30.75	119.00 ± 26.12	0.38	>0.99
Pneumoperitoneum Duration, min	78.24 ± 28.28	77.40 ± 27.76	82.88 ± 23.52	0.96	>0.99
Estimated bleeding, ml	58.33 ± 18.86	58.33 ± 18.86	58.33 ± 18.86	0.08	>0.99
Hospitalization Duration, day	8.95 ± 2.23	8.60 ± 2.05	9.12 ± 1.90	1.32	>0.99
PONV/ or motion sickness history, n (%)	14(33.3%)	16(38.1%)	9(21.4%)	–	>0.99
History of abdominal, n (%)	15(35.7%)	16(38.1%)	5(12%)	–	>0.99

Notes: Data are presented as mean ± SD for normally distributed continuous variables. One-way ANOVA, followed by Bonferroni post-hoc tests for pairwise comparisons. Data are presented as mean ± SD for normally distributed continuous variables. One-way ANOVA, followed by Bonferroni post-hoc tests for pairwise comparisons.Data are presented as n (%). Chi-square test or Fisher’s exact test was used for comparisons between groups. Bonferroni correction was applied for multiple comparisons (adjusted α = 0.0083 for four groups).

Abbreviations: -: Fisher’s exact test; Group L, low-dose group; Group M, medium-dose group; Group M, high-dose group.

Among the three groups, the incidence of nausea was significantly higher in the H group (*P* < 0.0038) (Table 2).

**Table 2:** The incidence of nausea and vomiting within 24 hours postoperatively.

Group	Nausea	Vomiting
Group L (n = 42)	8(19.0%)	4(9.5%)
Group M (n = 42)	7(16.7%)	3(7.1%)
Group H (n = 42)	18(42.9%)^ab^	8(19.0%)

Notes: Data are presented as n (%). Chi-square test or Fisher’s exact test was used for comparisons between groups. Bonferroni correction was applied for multiple comparisons (adjusted α = 0.0083 for four groups). Adjusted *P* < 0.0083 was considered statistically significant. Compared with group L, ^a^*P* < 0.0083; compared with group M, ^b^*P* < 0.0083.

**Fig 2:**
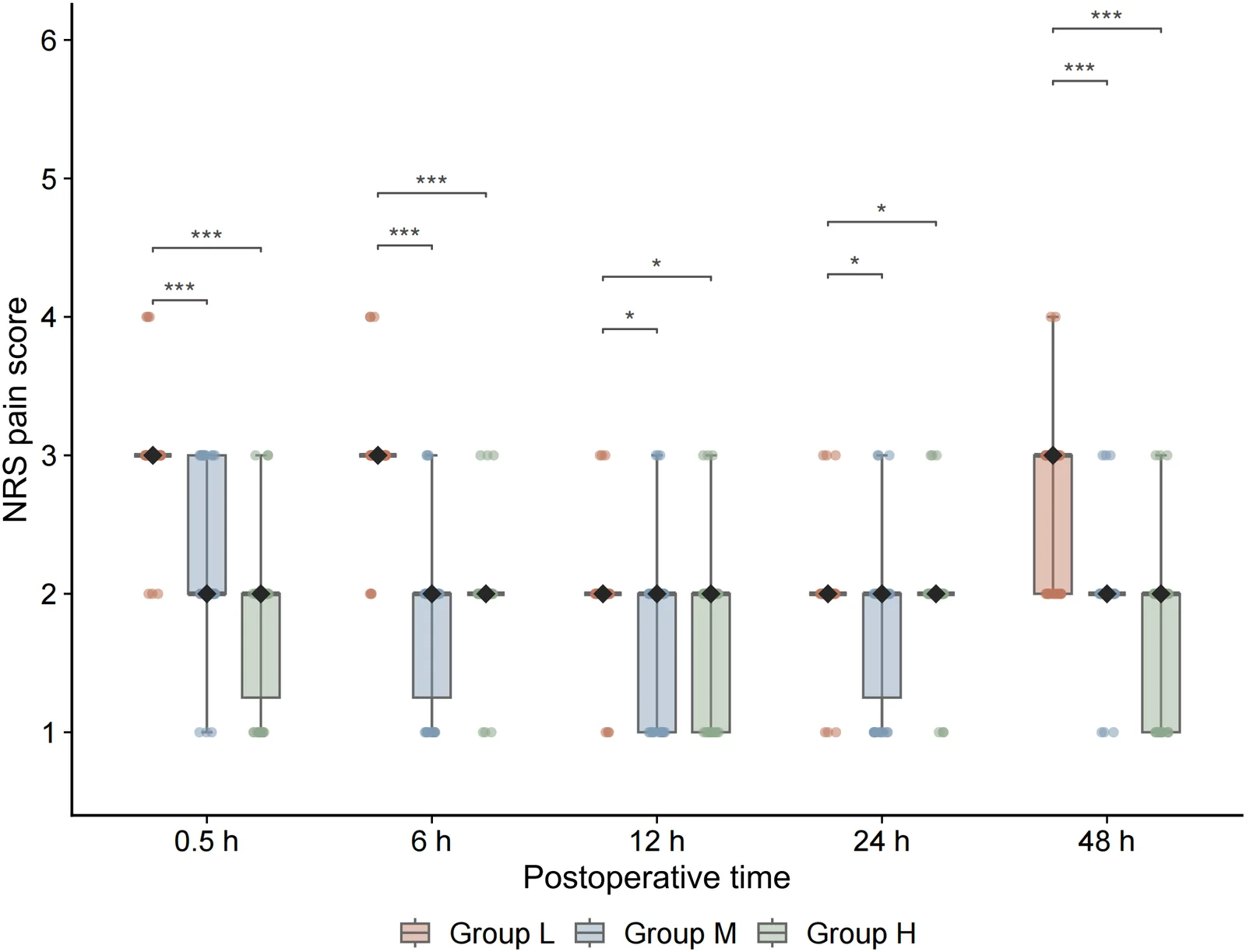
Comparison of NRS pain scores during postoperative coughing among Group L, Group M and Group H. Comparison of NRS pain scores during postoperative coughing among Group L (0.1 mg/kg oliceridine), Group M (0.35 mg/kg), and Group H (0.5 mg/kg). Boxes represent the median and interquartile range (IQR); whiskers extend to 1.5 × IQR; diamonds indicate the median; individual observations are shown as jittered points. *P < 0.0083, ***P < 0.00017.

Compared with Group L, Groups M and H showed significantly lower activity NRS scores at 0.5, 6, and 48 hours postoperatively (*P* < 0.00017). Significant reductions were also observed at 12 and 24 hours, albeit with a higher threshold (*P* < 0.0083) (Fig 2 and Table 3).

**Table 3:** Postoperative Numerical Rating Scale (NRS) Scores and Ramsay sedation scores.

Variables	Group	Quantity	0.5h	6h	12h	24h	48h
Rest NRS	Group L	42	2.0(2.0 ~ 2.0)^b^	2.0(2.0 ~ 2.0)^a^	2.0(2.0 ~ 2.0)^a^	1.5(1.0 ~ 2.0)^b^	1.0(1.0 ~ 2.0)^b^
	Group M	42	2.0(2.0 ~ 2.0)^b^	2.0(1.2 ~ 2.0)^b^	2.0(1.0 ~ 2.0)^b^	1.0(1.0 ~ 2.0)^b^	1.0(1.0 ~ 2.0)^b^
	Group H	42	2.0(2.0 ~ 2.0)^b^	2.0(1.2 ~ 2.0)^b^	1.0(1.0 ~ 2.0)^b^	1.0(1.0 ~ 2.0)ª	1.0(1.0 ~ 2.0)^b^
Cough NRS	Group L	42	3.0(3.0 ~ 3.0)^a^	3.0(3.0 ~ 3.0)^a^	2.0(2.0 ~ 2.0)^a^	2.0(2.0 ~ 2.0)^a^	3.0(2.0 ~ 3.0)^a^
	Group M	42	2.0(2.0 ~ 3.0)^b^	2.0(1.2 ~ 2.0)^b^	2.0(1.0 ~ 2.0)^b^	2.0(1.2 ~ 2.0)^b^	2.0(2.0 ~ 2.0)^b^
	Group H	42	2.0(1.2 ~ 2.0)^b^	2.0(2.0 ~ 2.0)^b^	2.0(1.0 ~ 2.0)^b^	2.0(2.0 ~ 2.0)^b^	2.0(1.0 ~ 2.0)^b^

Notes: Data are presented as median (Q1, Q3). Kruskal-Wallis H test was used for non-normally distributed continuous variables. Wilcoxon rank-sum test with Bonferroni correction was used for post-hoc pairwise comparisons. Groups sharing no common letter are significantly different. The significance level was set at *P* < 0.00017 for comparisons at 0.5h, 6h, and 48h, and at *P* < 0.0083 for comparisons at 12h and 24h.

There were no statistically significant differences in Ramsay sedation scores among the three groups at any postoperative time point (Table 4).

**Table 4:** Postoperative Ramsay sedation scores.

Variables	Group	Quantity	0.5h	6h	12h	24h	48h
Ramsay Sedation scores	Group L	42	2.0(2.0 ~ 2.0)^a^	2.0(2.0 ~ 2.0)^a^	2.0(2.0 ~ 2.0)^a^	2.0(2.0 ~ 2.0)^a^	2.0(2.0 ~ 2.0)^a^
	Group M	42	2.0(2.0 ~ 2.0)^a^	2.0(2.0 ~ 2.0)^a^	2.0(2.0 ~ 2.0)^a^	2.0(2.0 ~ 2.0)^a^	2.0(2.0 ~ 2.0)^a^
	Group H	42	2.0(2.0 ~ 2.0)^a^	2.0(2.0 ~ 2.0)^a^	2.0(2.0 ~ 2.0)^a^	2.0(2.0 ~ 2.0)^a^	2.0(2.0 ~ 2.0)^a^

Notes: There were no statistically significant differences in Ramsay sedation scores among the three groups at any postoperative time point (Table 3). This was confirmed by the Kruskal-Wallis test with Bonferroni correction, where all groups shared the same letter (a) across all time points, indicating no significant pairwise differences.

Compared with Group L, Group M and H showed significantly fewer effective and total pump presses (*P* < 0.0083). Compared with Group M, Group H had significantly fewer effective and total pump presses. There were no statistically significant differences in tramadol and metoclopramide usage rates among the three groups (Table 5).

**Table 5:** Use of the analgesic pump and remedial measures within 48 hours postoperatively.

Group	Quantity	Counts of PCIA effective activation, times	Counts of PCIA total activation, times	Postoperative tramadol consumption, n (%)	Postoperative Metoclopramide Consumption, n(%)
Group L	42	6.43 ± 1.23	7.67 ± 1.26	3(7%)	2(4.8%)
Group M	42	5.64 ± 1.14^a^	6.93 ± 1.28^a^	2(4.8%)	2(4.8%)
Group H	42	3.74 ± 1.15^ab^	5.31 ± 1.05^ab^	1(2.4%)	6(14.3%)

Notes: Data are presented as mean ± SD for normally distributed continuous variables. One-way ANOVA, followed by Bonferroni post-hoc tests for pairwise comparisons (assuming six comparisons in accordance with the original four-group framework). *P* < 0.0083 was considered statistically significant. Compared with group L, ª*P* < 0.0083, Compared with group M, ^b^ *P* < 0.0083.

Compared with the M group, the incidence of nausea was significantly higher in the H group (*P* < 0.0083). There were no statistically significant differences in the incidence of respiratory depression, dizziness among the three groups. No cases of skin itching or somnolence occurred (Table 6)

**Table 6:** Safety outcomes.

Group	Respiratory Depression	Dizziness	Constipation	Pruritus	Somnolence
Group L	0/42(0.0%)	2/41(4.9%)	8/42(19.0%)	0(0%)	0(0%)
Group M	0/42(0.0%)	1/41(2.4%)	5/42(11.9%)	0(0%)	0(0%)
Group H	1/42(2.4%)	2/42(4.8%)	7/42(16.7%)	0(0%)	0(0%)

Notes: Data are presented as n (%). Chi-square test or Fisher’s exact test was used for comparisons between groups. Bonferroni correction was applied for multiple comparisons (adjusted α = 0.0083 for four groups). Adjusted *P* < 0.0083 was considered statistically significant.

Compared with the M group, the L group and H group showed significantly prolonged time to first postoperative flatus and significantly lower QoR-15 scores at 24 and 48 hours postoperatively (Table 7).

**Table 7:** Recovery indicators.

Group	QoR-15, points			First Postoperative Flatus, h	First Postoperative Ambulation, h
	Preoperative	24 h postoperative	48 h postoperative		
Group L	131.62 ± 3.22^a^	101.64 ± 5.73^a^	110.17 ± 4.76^a^	22.30 ± 2.85^a^	24.08 ± 2.20^a^
Group M	131.19 ± 3.72^a^	106.52 ± 5.43^b^	115.98 ± 4.81^b^	18.12 ± 2.56^b^	20.32 ± 1.69^b^
Group H	130.69 ± 3.22^a^	103.12 ± 3.96^a^	109.07 ± 5.53^a^	20.22 ± 2.53^a^	23.08 ± 2.54^a^

Notes: Data are presented as mean ± SD for normally distributed continuous variables. One-way ANOVA followed by Bonferroni post-hoc tests for pairwise comparisons (assuming six comparisons in accordance with the original four-group framework). *P* < 0.0083 was considered statistically significant.

## Discussion

This prospective double-blind trial demonstrated significant differences in efficacy and safety profiles among the three oliceridine doses for patient-controlled analgesia (PCA) following laparoscopic total hysterectomy. Although the study was originally designed as a four-arm trial (including a sufentanil control arm), the premature termination of this arm limited the final analysis to the three oliceridine regimens. Key findings indicate that while medium- and high-dose Oliceridine demonstrated superior dynamic analgesic effects and reduced postoperative patient-controlled analgesia requirements compared to low-dose Oliceridine, the medium dose (0.35 mg/kg) achieved the optimal balance between analgesic efficacy and minimal side effects within the investigated dose range. This dose facilitates postoperative recovery, providing evidence-based guidance for clinical application [4,21]. The significant reduction in effective bolus doses and total bolus doses in both the medium-dose and high-dose groups further corroborates this finding, reflecting superior background analgesia and enhanced patient satisfaction with the analgesic regimen. This observation aligns with results from other studies on opioid medications used for PCIA [23].

The key distinction among the oliceridine-specific regimens was observed in recovery outcomes. Although the high-dose group further reduced the number of additional doses administered, this marginal benefit came at a significant cost: the time to first flatus was significantly prolonged in the high-dose group compared to the medium-dose group, and time to ambulation showed a similar trend. This suggests a potential “optimal dose range” for Oliceridine—one that provides sufficient analgesia to promote activity without inducing significant opioid-induced bowel dysfunction (OIBD). In contrast, the high dose markedly prolonged time to first flatus, a hallmark manifestation of OIBD—a common and distressing complication among μ-opioid receptor agonists [24]. While relieving pain, the medium dose does not excessively suppress gastrointestinal motility and motor function—both critical factors influencing early postoperative recovery [25]. This finding is crucial, as accelerating gastrointestinal function recovery is a core objective of Enhanced Recovery After Surgery (ERAS) protocols [26].

Notably, the high-dose oliceridine group (0.5 mg/kg) exhibited a significantly higher incidence of nausea compared to the medium- and low-dose groups. This dose-dependent escalation suggests that even with a G protein-biased agonist, higher doses may engage β-arrestin-mediated pathways sufficiently to elicit typical opioid-related side effects. This observation aligns with the concept that ‘biased agonism’ is not an absolute on/off phenomenon but a matter of relative pathway selectivity. It underscores the importance of identifying the therapeutic window within which G-protein signaling predominates—a window that appears to be optimally maintained at the 0.35 mg/kg dose in this surgical population [27]. No significant differences were observed in Ramsay sedation scores or rescue medication doses of tramadol and metoclopramide across groups. These findings indicate that low doses were insufficient for dynamic pain management, while moderate and high doses, though not causing excessive sedation, demonstrated varying gastrointestinal tolerability. Group M demonstrated superior QoR-15 scores and patient satisfaction at both 24 and 48 hours compared to other groups, whereas Group H showed a significant decline in these measures after 48 hours. This composite endpoint data strongly supports the intermediate-dose regimen. The QoR-15 scale is a validated assessment tool that comprehensively measures multidimensional postoperative recovery, including physical comfort, functional independence, and nausea/vomiting relief [13]. The Group M results align with the core objective of the ERAS protocol, which emphasizes not only adequate analgesia but also the rapid and comfortable return of patients to baseline functional status. Furthermore, the absence of significant differences in Ramsay sedation scores indicates that Oliceridine possesses a wide safety margin within the 0.1–0.5 mg/kg dosage range, avoiding excessive sedation. This finding is consistent with previous studies reporting its favorable sedation safety profile [28].

This study has several limitations: First, the sample size was relatively small (135 cases), and the study population was restricted to patients aged 18–65 years with a BMI of 18.5 ~ 35.0 kg/m² and ASA I–II status, potentially limiting the generalizability of the results to elderly patients or those with severe comorbidities. Second, the study was conducted at a single center and focused on a specific surgical population (laparoscopic hysterectomy), limiting the generalizability of its conclusions to other surgical types or patient groups. Third, the observation period was restricted to 48 hours, failing to assess long-term outcomes or risks of chronic postoperative pain. Finally, the fixed-dose regimen was not titrated according to individual response, which may have influenced the final results.

In summary, for patients undergoing laparoscopic total hysterectomy, the PCIA dose of oliceridine 0.35 mg/kg appears optimal within the studied dosage range. This dose provides effective dynamic analgesia, reduces the need for rescue medication, and compared to lower and higher doses, it decreases the incidence of postoperative opioid-related adverse events (ORAEs) while accelerating gastrointestinal function recovery. These advantages translate into quantifiable improvements in early postoperative recovery quality and higher patient satisfaction. Future research should focus on comparing oliceridine with other first-line analgesics and exploring its value within multimodal analgesia regimens.

## Conclusion

Within the investigated dose range of oliceridine for PCIA, the 0.35 mg/kg dose appears optimal for laparoscopic total hysterectomy. This dose provides effective dynamic pain control while minimizing nausea and accelerating gastrointestinal recovery, representing the best balance between efficacy and safety among the tested oliceridine regimens.

## Supporting information

S1 Checklist CONSORT 2025 checklist of information to include when reporting a randomized trial*. (DOCX)

S1 File Study protocol. (DOCX)
