# Supplementary material for: Efficacy of different doses of oliceridine for Patient-Controlled Intravenous Analgesia in patients undergoing Total Laparoscopic Hysterectomy
Source: PLoS One. 2026 Sep 3;21(9):e0357372. doi: 10.1371/journal.pone.0357372 (PMC13541144; doi:10.1371/journal.pone.0357372)
Supplement: Supplementary file 1 [file pone.0357372.s001.docx]

|  | Section/topic | No | CONSORT 2025 checklist item description | Reported on page no. |
| --- | --- | --- | --- | --- |
|  | **Title and abstract** | | |  |
|  | Title and structured abstract | 1a | Efficacy of Different Doses of Oliceridine for Patient-Controlled Intravenous Analgesia in Patients Undergoing Total Laparoscopic Hysterectomy. | 1 |
|  |  | 1b | Objective  To evaluate the efficacy of different doses for oliceridine for patient-controlled analgesia during total laparoscopic hysterectomy.  Methods  Patients were randomized to receive a 48-hour postoperative PCIA with oliceridine at one of three doses: 0.1, 0.35, or 0.5 mg/kg. Each dose was diluted to a total volume of 100 ml with normal saline. The following outcomes were recorded: NRS pain scores (at rest and during cough), Ramsay sedation scores, incidence of rescue analgesia, various recovery indicators, and opioid-related adverse events.  Results  Compared to the low-dose group, the moderate- and high-dose groups demonstrated significant improvements in cough-pain scores and bolus press attempts (p < 0.05). Further comparison revealed that the moderate-dose group showed the best recovery outcomes, including shorter time to first flatus and ambulation, and higher QoR-15 scores. In contrast, although the high-dose group provided more potent analgesia, it was associated with a higher incidence of nausea, delayed flatus, and a significantly lower quality of recovery (QoR-15) score at 48 hours postoperatively compared to the moderate-dose group (p < 0.05). | 1-2 |
|  | **Open science** | | |  |
|  | Trial registration | 2 | Chinese Clinical Trial Registration Center (ChiCTR2500097470, February 19, 2025). | 5 |
|  | Protocol and statistical analysis plan | 3 | The trial protocol and statistical analysis plan are available in the Supplementary Material. | 10 |
|  | Data sharing | 4 | YES |  |
|  | Funding and conflicts of interest | 5a | Self-funded by the researchers. |  |
|  |  | 5b | The authors declare that they have no known competing financial interests or personal relationships that could have appeared to influence the work reported in this paper. |  |
|  | **Introduction** | | |  |
|  | Background and rationale | 6 | Traditional opioids used for postoperative analgesia can cause opioid-related adverse effects, which impact the quality of patient recovery. | 2 |
|  | Objectives | 7 | The novel analgesic oliceridine can effectively reduce opioid-related adverse effects, providing a superior option for postoperative analgesia in patients undergoing laparoscopic total hysterectomy. | 1 |
|  | **Methods** | | |  |
|  | Patient and public involvement | 8 | No patient or public involvement occurred in the design, conduct, or reporting of this trial. |  |
|  | Trial design | 9 | Participants were then allocated in a 1:1:1 ratio to either the Group L、group M or Group H. | 5 |
|  | Changes to trial protocol | 10 | No changes were made to the trial protocol after commencement of recruitment. |  |
|  | Trial setting | 11 | Ethics Committee of the First Affiliated Hospital of Shihezi University (No. KJ-2024-514-02, 2024-12-10) | 5 |
|  | Eligibility criteria | 12a | American Society of Anesthesiologists (ASA) I-II; aged 18-65 years; understanding of the study and signing of informed consent. | 5 |
|  | Intervention and comparator | 13 | After extubation, all three groups received a loading dose of 1.5 mg oliceridine intravenously, with oxygen supplied via a mask at a flow rate of 5 L/min. Oxygen administration ceased after 5 minutes, and postoperative analgesia was initiated.  Patients in the group L received PCIA: 0.1 mg/kg oliceridine dissolved in 100 mL saline (baseline rate 2 mL/h, bolus 0.5 mL, lockout 15 min); Group M patients received PCIA: 0.35 mg/kg oliceridine dissolved in 100 mL saline (baseline rate 2 mL/h, bolus 0.5 mL, lockout 15 min); Group H patients received PCIA: 0.5 mg/kg oliceridine dissolved in 100 mL saline (baseline rate 2 mL/h, bolus 0.5 mL, lockout 15 min). | 8 |
|  | Outcomes | 14 | The primary endpoints were NRS pain scores and Ramsay sedation scores at 0.5, 6, 12, 24, and 48 hours post-loading dose during rest and coughing.Pain rescue utilization : Recorded effective pump presses, total pump presses, and tramadol usage within 48 hours post-operative. Postoperative recovery quality assessed at 24 and 48 hours using the validated 15-item Quality of Recovery (QoR-15) scale (total score range 0–150; higher scores indicate better postoperative recovery).Adverse events at 48 hours postoperatively, including nausea, vomiting, respiratory depression, dizziness, pruritus, and constipation (failure to defecate within 48 hours) | 9 |
|  | Harms | 15 | Adverse events were collected based on spontaneous reports from participants or observations by clinical investigators during routine follow-up visits, without systematic active monitoring. This approach was used because oliceridine has been widely used in clinical practice with a well-established safety profile, and the study primarily focused on efficacy endpoints. | 9 |
|  | Sample size | 16a | Sample size was calculated using PASS15 software. In the pilot study, the NRS pain scores during activity at 24 hours postoperatively were (4.1 ± 1.1) points, (2.5 ± 0.8) points, and (2.0 ± 0.4) points for the L, M, and H groups, respectively. With α = 0.05 and 1-β = 0.8, the calculated sample size was 108 participants for one-way ANOVA. Considering a 20% dropout rate, 135 patients needed to be enrolled. | 10 |
|  |  | 16b | No interim analyses were conducted. |  |
|  | Randomisation: |  |  |  |
|  | Sequence generation | 17a | Randomization was performed using SPSS software (version 25.0). | 10 |
|  | Allocation concealment mechanism | 18 | A random number table method was employed, assigning each patient a random number in a 1:1:1 ratio based on their enrollment sequence. An independent statistician outside the research team retrieved the random number table according to the enrollment order, generated the grouping results, and sealed them in envelopes. | 10 |
|  | Implementation | 19 | Recruiting physicians were solely responsible for collecting and submitting patient information without access to the allocation results. The independent statistician placed the allocation information (including group assignment, intervention, and medication preparation method) into an opaque, sealed envelope and arranged the envelopes in sequence. On the day of surgery, a blinded medication administrator—uninvolved in any study procedures (including medication preparation, anesthesia, or follow-up)—will opened the envelopes. Based on the grouping information, they will provide the corresponding numbered study medication (pre-prepared by the pharmacy according to the randomized protocol, with identical appearance) to the medication preparer. The medication preparer only verify the patient ID and remain unaware of the drug's assigned group or intervention. They prepare the required study drug based on the grouping information in the envelope and deliver it to the anesthesiologist. The drug is labeled as “Study Drug” with the patient ID. | 6 |
|  | Blinding | 20a | Patients, anesthesiologist, surgeon, postoperative follow-up personnel and data collectors | 6 |
|  | Statistical methods | 21 | Statistical analysis was performed using SPSS 26.0 software. Normally distributed continuous data are presented as mean ± standard deviation (x̄ ± SD). Inter-group comparisons were conducted using one-way analysis of variance (ANOVA), with post analysis employing LSD-t tests and SNK tests. Non-normally distributed continuous data were expressed as median and quartile range [M(Q1, Q3)]. Inter-group comparisons were performed using the Kruskal-Wallis test, with pairwise comparisons using the Bonferroni-corrected Wilcoxon signed-rank test. Qualitative data were expressed as cases (%) and compared between groups using the χ² test or Fisher's exact test. P < 0.05 indicated statistically significant differences. | 10 |
|  | **Results** | | |  |
|  | Participant flow, including flow diagram | 22 | This study initially recruited 135 patients. In the L group, 2 patients failed to use the analgesic pump correctly; in the M group, 1 patient used the analgesic pump incorrectly and 1 patient had a surgical duration exceeding 3 hours; in the H group, 1 patient had a surgical duration exceeding 3 hours and 1 patient was transferred to the ICU postoperatively. These patients have been excluded, resulting in a final sample size of 126 patients.   | 10 |
|  | Recruitment | 23 | First Affiliated Hospital of Shihezi University between February 2025 and April 2026 | 5 |
|  | Intervention and comparator delivery | 24a | After extubation, all three groups received a loading dose of 1.5 mg oliceridine intravenously, with oxygen supplied via a mask at a flow rate of 5 L/min. Oxygen administration ceased after 5 minutes, and postoperative analgesia was initiated.  Patients in the group L received PCIA: 0.1 mg/kg oliceridine dissolved in 100 mL saline (baseline rate 2 mL/h, bolus 0.5 mL, lockout 15 min); Group M patients received PCIA: 0.35 mg/kg oliceridine dissolved in 100 mL saline (baseline rate 2 mL/h, bolus 0.5 mL, lockout 15 min); Group H patients received PCIA: 0.5 mg/kg oliceridine dissolved in 100 mL saline (baseline rate 2 mL/h, bolus 0.5 mL, lockout 15 min). | 6 |
|  | Baseline data | 25 | **Table 1  **Comparison of Baseline Characteristics Between Groups**** **Variables** \| **Group L (n=42)** \| **Group M (n=42)** \| **Group H (n=42)**; Age,yr \| 52.79±5.54 \| 52.79±5.54 \| 50.57±7.25; Height, m \| 1.59±0.05 \| 1.59±0.05 \| 1.59±0.05; Weight, kg \| 63.19±5.33 \| 63.67±6.64 \| 63.12±5.70; BMI, kg/㎡ \| 24.99±1.91 \| 25.25±2.49 \| 24.98±2.25; Apfel \| 3.38±0.49 \| 3.38±0.49 \| 3.14±0.52; Operation duration, min \| 109.79±29.91 \| 110.52±30.75 \| 119.00±26.12; Pneumoperitoneum Duration, min \| 78.24±28.28 \| 77.40±27.76 \| 82.88±23.52; Estimated bleeding, ml \| 58.33±18.86 \| 58.33±18.86 \| 58.33±18.86; Hospitalization Duration, day \| 8.95±2.23 \| 8.60±2.05 \| 9.12±1.90; PONV/ or motion sickness history, n (%) \| 14（33.3%） \| 16(38.1%) \| 9(21.4%); History of abdominal, n (%) \| 15（35.7%） \| 16(38.1%) \| 5(12%) | 10 |
|  | Numbers analysed,  outcomes and estimation | 26 | For each primary and secondary outcome, by group:  ● NRS pain scores and Ramsay sedation scores at 0.5, 6, 12, 24, and 48 hours  ● Pain rescue utilization  ●Recovery indicators  ● Safety | 12  10  11 |
|  | Harms | 27 | NO |  |
|  | Ancillary analyses | 28 | NO |  |
|  | **Discussion** | | |  |
|  | Interpretation | 29 | or patients undergoing laparoscopic total hysterectomy, the PCIA dose of oliceridine 0.35 mg/kg appears optimal within the studied dosage range. This dose provides effective dynamic analgesia, reduces the need for rescue medication, and compared to lower and higher doses, it decreases the incidence of postoperative opioid-related adverse events (ORAEs) while accelerating gastrointestinal function recovery. These advantages translate into quantifiable improvements in early postoperative recovery quality and higher patient satisfaction. Future research should focus on comparing oliceridine with other first-line analgesics and exploring its value within multimodal analgesia regimens. | 15 |
|  | Limitations | 30 | This study has several limitations: First, the sample size was relatively small (135 cases), and the study population was restricted to patients aged 18–65 years with a BMI of 18.5～35.0 kg/m² and ASA I–II status, potentially limiting the generalizability of the results to elderly patients or those with severe comorbidities. Second, the study was conducted at a single center and focused on a specific surgical population (laparoscopic hysterectomy), limiting the generalizability of its conclusions to other surgical types or patient groups. Third, the observation period was restricted to 48 hours, failing to assess long-term outcomes or risks of chronic postoperative pain. Finally, the fixed-dose regimen was not titrated according to individual response, which may have influenced the final results. | 16 |
