# Supplementary material for: Efficacy of different doses of oliceridine for Patient-Controlled Intravenous Analgesia in patients undergoing Total Laparoscopic Hysterectomy
Source: PLoS One. 2026 Sep 3;21(9):e0357372. doi: 10.1371/journal.pone.0357372 (PMC13541144; doi:10.1371/journal.pone.0357372)
Supplement: Supplementary file 2 [file pone.0357372.s002.docx]

**Study Protocol**

(Version Number: 2.0 Release Date: December 8, 2024)

Project Title: Effect of Oliceridine on Postoperative Nausea and Vomiting in Patients Undergoing Laparoscopic Total Hysterectomy Principal

Investigator: Deng Jiaojiao

Contact Phone Number: +86-133-8512-8576

Study Period: December 2024 - October 2025

****Study Protocol****

****Study Objective:**** To demonstrate that, compared to sufentanil, the application of oliceridine in patients undergoing laparoscopic total hysterectomy can provide effective analgesia while reducing the incidence of nausea and vomiting, thereby offering a reference basis for the clinical use of oliceridine in anesthesia.

****Study Design:**** Randomized, double-blind, controlled trial.

****Sample Size:**** A total of 216 cases, comprising 162 cases in the experimental group and 54 cases in the control group. (Sample size was calculated using PASS 15.0 software. Literature review indicated vomiting incidence rates for oliceridine as 0.1 mg (20%), 0.35 mg (30%), and 0.5 mg (42%), respectively. The primary outcome measure of this study is the incidence of postoperative nausea and vomiting within 24 hours. Assuming α = 0.05 and 1-β = 0.8, and accounting for a 20% dropout rate, 54 patients are planned to be enrolled in each group.)

****Source of Study Subjects:**** Patients scheduled for elective laparoscopic total hysterectomy at The First Affiliated Hospital of Shihezi University.

****Treatment/Observation Period:**** 2024 to 2025.

****Inclusion Criteria:****

Aged 18 to 65 years;

American Society of Anesthesiologists (ASA) physical status classification I-II;

Able to cooperate with the completion of various assessment scales and provide signed informed consent.

**Exclusion Criteria:**

History of allergy to anesthetic drugs or contraindications to opioids;

Patients with severe respiratory depression;

Known or suspected gastrointestinal obstruction;

Presence of neurological disorders, or long-term use of hypnotics or sedatives;

Moderate to severe hypertension and/or patients for whom a significant blood pressure increase would be life-threatening;

Patients with acute or chronic pain conditions;

Severe hepatic or hepatorenal dysfunction;

Refusal to sign the informed consent form or uncooperative patients.

Treatment/Observation Protocol:The incidence of nausea and vomiting within 24 hours postoperatively will be recorded for each patient

****Experimental Groups:**** Postoperative intravenous patient-controlled analgesia (PCA) with oliceridine at doses of 0.1 mg/kg, 0.35 mg/kg, and 0.5 mg/kg, respectively.

****Control Group:**** Postoperative intravenous PCA with sufentanil at a dose of 2 μg/kg.

****R**emarks:** None.

**Evaluation Indicators:**

Occurrence of nausea and vomiting.

**Effectiveness Evaluation Indicators:**

Primary Efficacy Endpoint: Incidence of postoperative nausea and vomiting (PONV).

Secondary Efficacy Endpoint: Frequency of postoperative rescue antiemetic medication use.

****Study Duration:**** It is anticipated that this clinical trial will be completed within 12 months after the funding is secured.

****Study Protocol Main Text****

1. ****Research Background****

In 1997, Danish surgeons Wilmore and Kehlet proposed the theory of Enhanced Recovery After Surgery (ERAS). This theory emphasizes the optimization of perioperative management measures based on multidisciplinary collaboration and evidence-based medicine, aiming to reduce various stress responses, alleviate postoperative pain, and promote the rapid recovery of organ functions. The ultimate goals are to alleviate patients' psychological trauma, reduce complications, shorten hospital stays, lower the risks of readmission and mortality, and decrease medical costs. With the rapid development of medical technology, the ERAS concept has been widely promoted and applied, and it is currently recommended in guidelines related to colorectal surgery, hepatobiliary surgery, gynecology, and other fields.

Laparoscopic surgery, known for its minimal trauma, reduced stress response, and rapid postoperative recovery, is now widely used in the treatment of gynecological diseases. Examples include ovarian cystectomy, adnexal tumor resection, myomectomy, and hysterectomy. Total hysterectomy is the most common surgical procedure for non-pregnant women, with approximately 2.8 million patients undergoing this surgery annually in China. Although laparoscopic surgery is often considered to have better postoperative recovery prospects due to its minimally invasive nature, 50% of patients experience postoperative nausea (PON), and 30% experience postoperative vomiting (POV). Additionally, CO₂ pneumoperitoneum, traction during surgery, pain stimulation, and the use of opioids can also lead to an increased incidence of postoperative nausea and vomiting (PONV) in patients. PONV affects patient satisfaction with their postoperative recovery and may even cause electrolyte imbalances, wound dehiscence, and other complications, ultimately leading to delayed recovery, prolonged hospital stays, and increased medical costs.

Traditional opioids used during the perioperative period also have significant adverse effects, such as nausea, vomiting, constipation, chronic pain, and drug dependence, which markedly impact the quality of patient recovery. Sufentanil is widely used in clinical practice for general anesthesia induction, intraoperative maintenance, and postoperative analgesia due to its potent analgesic properties and favorable hemodynamic stability. However, high doses of sufentanil may cause respiratory depression. Although both laparoscopic procedures and gynecological surgeries have been identified as independent risk factors for PONV, the frequent need for opioids—in addition to female gender—increases the risk of PONV in women undergoing these surgeries to approximately 80%. Although PONV is not a life-threatening side effect, it can significantly affect treatment outcomes, including the patient’s quality of life. Furthermore, ineffective management of postoperative pain can also impair functional recovery, prolong hospital stays, and increase medical costs. Therefore, the role of anesthesia should not be confined to merely meeting surgical requirements. Perioperative anesthesia management protocols should pursue multiple objectives, including alleviating preoperative anxiety, reducing intraoperative stress responses, optimizing respiratory and circulatory management to create optimal surgical conditions, and actively preventing and treating postoperative complications.

2. **Study Objectives**

This study aims to demonstrate that, compared to sufentanil, oliceridine provides effective analgesia and reduces the incidence of nausea and vomiting in patients undergoing laparoscopic total hysterectomy, thereby offering a reference basis for the clinical application of oliceridine in anesthesia.

3. **Research Methods**

Study Subjects Patients scheduled for elective laparoscopic total hysterectomy at The First Affiliated Hospital of Shihezi University between December 2024 and October 2025 will be enrolled in this study after providing informed consent.

****3.1 Inclusion Criteria****① Aged 18 to 65 years;② American Society of Anesthesiologists (ASA) physical status classification I-II;③ Able to cooperate with the completion of various assessment scales and provide signed informed consent.

****3.2 Exclusion Criteria****① Patients with a history of allergy to anesthetic drugs or contraindications to opioids;② Patients with severe respiratory depression;③ Known or suspected gastrointestinal obstruction;④ Patients with neurological disorders, or long-term use of hypnotics or sedatives;⑤ Patients with moderate to severe hypertension and/or for whom a significant increase in blood pressure would be life-threatening;⑥ Patients with acute or chronic pain conditions;⑦ Patients with severe hepatic or hepatorenal dysfunction;⑧ Patients who refuse to sign the informed consent form or are uncooperative.

****3. Grouping Method****

Using a random number table method with a 1:1:1:1 allocation ratio, each patient will be assigned a random number according to their enrollment sequence. Based on the generated random numbers, enrolled patients will be divided into groups. Grouping information (including group assignment, intervention measures, and drug preparation methods) will be sealed in opaque, sealed envelopes. These envelopes will be arranged in order and handed to a medication preparer on the day of surgery who is not involved in the anesthesia process or postoperative follow-up. The preparer will configure the required study drugs according to the grouping information in the envelope and deliver them to the anesthesiologist. The drugs will be labeled as "Study Drug" with the patient identification number. The two drug solutions will be indistinguishable in appearance. Throughout the process, the patient, anesthesiologist, surgeon, and postoperative follow-up personnel will be blinded to the group assignments.

****3.1 Anesthesia Protocol****

During the preoperative visit, patients enrolled in the trial will be guided to complete the Apfel score form. The planned anesthesia method, trial procedures, safety, risks, and precautions will be explained to the patient and their family. After obtaining their understanding and cooperation, informed consent will be acquired, and the informed consent form will be signed.Patients will routinely fast for 8 hours and abstain from fluids for 4 hours preoperatively. Upon entering the operating room, standard monitoring will be established, including electrocardiography (ECG), non-invasive blood pressure, heart rate, and oxygen saturation. After preparing emergency medications and equipment, general anesthesia induction will be administered. Following preoxygenation via face mask, the following intravenous drugs will be administered sequentially: midazolam 0.05 mg/kg, etomidate 0.2 mg/kg, sufentanil citrate 0.5 µg/kg, and rocuronium bromide 0.6 mg/kg. Orotracheal intubation will be performed once the patient loses consciousness, the eyelash reflex disappears, and jaw relaxation is observed. After successful intubation, mechanical ventilation will be initiated. Ventilator settings will be adjusted based on end-tidal carbon dioxide (PETCO2) to maintain PETCO2 between 35 and 45 mmHg.Anesthesia depth will be controlled using target-controlled infusion of propofol at 4-6 mg/kg/h and remifentanil at 0.15-0.30 μg/kg/min, maintaining the Bispectral Index (BIS) between 40 and 60. Simultaneously, based on surgical requirements, cisatracurium besylate will be continuously infused at a rate of 1-2 μg/kg/min to maintain neuromuscular blockade. Throughout the surgery, blood pressure will be maintained within 20% of the baseline measurement by adjusting the doses of intravenously infused medications according to BIS values. If hemodynamic variables deviate by more than 20% from the baseline measurement, intravenous administration of vasoactive drugs will be initiated immediately. **Group S:** A loading dose of 10 µg sufentanil was administered 30 minutes before the end of surgery. The analgesic pump formula was: sufentanil 2 µg/kg diluted to 100 ml. **Group A1:** A loading dose of 2 mg oliceridine was administered 5 minutes before the end of surgery. The analgesic pump formula was: oliceridine 0.1 mg/kg diluted to 100 ml. **Group A2:** A loading dose of 2 mg oliceridine was administered 5 minutes before the end of surgery. The analgesic pump formula was: oliceridine 0.35 mg/kg diluted to 100 ml. **Group A3:** A loading dose of 2 mg oliceridine was administered 5 minutes before the end of surgery. The analgesic pump formula was: oliceridine 0.5 mg/kg diluted to 100 ml. The infusion of propofol was discontinued at the commencement of skin suturing, and the remifentanil infusion was ceased immediately upon completion of skin closure. At the conclusion of the surgery, 4 mg of ondansetron was administered intravenously for prophylactic antiemesis. Owing to the distinct pharmacokinetic properties of the medications utilized in the respective trial groups, all patients were transferred to the Post-Anesthesia Care Unit (PACU) for recovery and extubation. Tracheal extubation was performed after confirmation of the return of consciousness (ROC), defined as a response to verbal commands and the presence of adequate spontaneous respiration. In instances of inadequate analgesia within the PACU, 1 mg of but orphanol tartrate was administered. Respiratory depression was presumed if the respiratory rate fell below 6 breaths per minute or if the SpO₂ decreased below 90%. During data collection and assessment (in the PACU and at 24 hours postoperatively), an anesthesiologist who was blinded to the study group assignments inquired of the patients regarding the occurrence of adverse effects such as abdominal pain, abdominal distension, nausea, vomiting, pruritus, headache, dizziness, and visual disturbances.

****3.1.1Observation Indicators3.6.1 General Data****

Record the age, height, weight, BMI, ASA classification, smoking status, history of PONV and motion sickness, history of abdominal surgery, and the Apfel score from the day before surgery for patients in both groups.

****3.1.2 Primary Observation Indicators****

(1) Incidence of postoperative nausea and vomiting (PONV)

****3.1.3 Secondary Outcome Measures****

(1) Vital signs at various intraoperative time points: systolic blood pressure, diastolic blood pressure, mean arterial pressure (MAP), and heart rate (HR) at the following time points:

Before anesthesia induction (T1)

After anesthesia induction, before tracheal intubation (T2)

After tracheal intubation (T3)

Before skin incision (T4)

After skin incision (T5)

End of surgery (T6)

At extubation (T7)

(2) Intraoperative drug usage:

Propofol (mg)

Remifentanil (µg)

Ephedrine (mg)

Atropine (mg)

(3) Frequency and dosage of analgesic rescue medication in the PACU and ward.
(4) Number of effective PCA pump presses postoperatively, postoperative VAS score, and Ramsay sedation score.
(5) Postoperative adverse reactions: dizziness, headache, nausea, vomiting, abdominal pain, abdominal distension, delirium, nightmares, restlessness, hallucinations, diplopia.

(6) Time to first ambulation postoperatively (hours), time to first flatus postoperatively (hours), and length of hospital stay (days).

****3.2 Data Analysis****

The collected data will be entered into Excel and statistically analyzed using SPSS Statistics 27.0 software. Normally distributed measurement data will be expressed as mean ± standard deviation (x̄ ± s). Intergroup comparisons will be performed using one-way analysis of variance (ANOVA), with pairwise comparisons conducted using Bonferroni correction for post-hoc analysis. Intragroup comparisons will be performed using repeated measures analysis of variance. Non-normally distributed measurement data will be expressed as median and interquartile range [M (IQR)], and intergroup comparisons will be performed using the Kruskal-Wallis test. Count data will be expressed as number of cases (%), and intergroup comparisons will be performed using the chi-square test or Fisher's exact probability method. A P-value < 0.05 will be considered statistically significant.

4. ****Research Procedures****

Patients scheduled for elective laparoscopic total hysterectomy at the First Affiliated Hospital of Shihezi University between December 2024 and October 2025 will be enrolled in this study after providing informed consent. Using a random number table method with a 1:1:1:1 allocation ratio, each patient will be assigned a corresponding random number based on their enrollment sequence. Patients will be grouped according to the generated random numbers. The grouping information (including group assignment, intervention measures, and drug preparation methods) will be sealed in opaque, sealed envelopes. These envelopes will be arranged in order and handed to a medication preparer on the day of surgery who is not involved in the anesthesia process or postoperative follow-up. The preparer will configure the required study drugs according to the grouping information in the envelopes and deliver them to the anesthesiologist. The drugs will be labeled as "Study Drug" along with the patient identification number. The two drug solutions will be visually indistinguishable. Throughout the entire process, patients, anesthesiologists, surgeons, and postoperative follow-up personnel will be blinded to the group assignments.

During the preoperative visit, patients enrolled in this trial will be guided to complete the Apfel score form. The planned anesthesia method, along with the trial's procedures, safety considerations, potential risks, and necessary precautions, will be explained to the patient and their family. After obtaining their understanding and cooperation, and upon acquiring informed consent from both the patient and their family, the informed consent form will be signed.Patients will routinely fast for 8 hours and abstain from fluids for 4 hours preoperatively. Upon entering the operating room, standard monitoring, including electrocardiography (ECG), blood pressure, heart rate, and oxygen saturation, will be established. After emergency medications and equipment are prepared, general anesthesia induction will be initiated. Following preoxygenation with a facemask, the following drugs will be administered intravenously in sequence: midazolam 0.05 mg/kg, etomidate 0.2 mg/kg, sufentanil citrate 0.5 μg/kg, and rocuronium bromide 0.6 mg/kg. Orotracheal intubation will be performed once the patient loses consciousness, the eyelash reflex disappears, and jaw relaxation is observed. After successful intubation, mechanical ventilation will be commenced. Ventilator settings will be adjusted based on the end-tidal carbon dioxide (PetCO₂) level to maintain it between 35 and 45 mmHg. Anesthesia depth will be maintained using target-controlled infusion of propofol at 4-6 mg/kg/h and remifentanil at 0.15-0.30 μg/kg/min, with the goal of keeping the Bispectral Index (BIS) between 40 and 60. Simultaneously, cisatracurium besylate will be continuously infused at a rate of 1-2 μg/kg/min to maintain neuromuscular blockade as required by the surgical procedure. Throughout the operation, blood pressure will be maintained within 20% of the baseline measurement by adjusting the dosage of intravenously infused medications based on the BIS values. Should any hemodynamic variables deviate by more than 20% from the baseline measurement, intravenous administration of vasoactive drugs will be initiated immediately.Group S: A loading dose of 10 μg of sufentanil will be administered 30 minutes before the end of surgery. The patient-controlled analgesia (PCA) pump formula will be: sufentanil 2 μg/kg diluted to 100 ml.Group A1: A loading dose of 2 mg of oliceridine will be administered 5 minutes before the end of surgery. The PCA pump formula will be: oliceridine 0.1 mg/kg diluted to 100 ml.Group A2: A loading dose of 2 mg of oliceridine will be administered 5 minutes before the end of surgery. The PCA pump formula will be: oliceridine 0.35 mg/kg diluted to 100 ml.Group A3: A loading dose of 2 mg of oliceridine will be administered 5 minutes before the end of surgery. The PCA pump formula will be: oliceridine 0.5 mg/kg diluted to 100 ml.The propofol infusion will be discontinued upon initiation of skin suturing, and the remifentanil infusion will be ceased immediately upon completion of skin closure. At the conclusion of the surgery, 4 mg of ondansetron will be administered intravenously for prophylactic antiemesis. Due to the specific pharmacokinetic properties of the drugs used in the respective trial groups, all patients will be transferred to the Post-Anesthesia Care Unit (PACU) for recovery and extubation. Tracheal extubation will be performed after confirmation of return of consciousness (ROC), defined as responsiveness to verbal commands and the presence of adequate spontaneous respiration. In cases of insufficient analgesia in the PACU, 1 mg of butorphanol tartrate will be administered. Respiratory depression will be presumed if the respiratory rate falls below 6 breaths per minute or if SpO₂ decreases below 90%. During data collection and assessment (in the PACU and at 24 hours postoperatively), an anesthesiologist who is blinded to the study group assignments will inquire whether patients have experienced adverse reactions such as abdominal pain, abdominal distension, nausea, vomiting, pruritus, headache, dizziness, and visual disturbances.

The collected data will be entered into Excel and statistically analyzed using SPSS 27.0 software. Normally distributed measurement data will be expressed as mean ± standard deviation (x̄ ± s). Intergroup comparisons will be performed using one-way analysis of variance (ANOVA), with pairwise comparisons conducted using Bonferroni correction for post-hoc analysis. Intragroup comparisons will be performed using repeated measures analysis of variance. Non-normally distributed measurement data will be expressed as median and interquartile range [M (IQR)], and intergroup comparisons will be performed using the Kruskal-Wallis test. Count data will be expressed as number of cases (percentage), and intergroup comparisons will be performed using the chi-square test or Fisher's exact probability method. A p-value < 0.05 will be considered statistically significant.

5. ****Benefits and Risks Potential benefits****

That participants may gain from this clinical trial include achieving comparable analgesic effects while simultaneously reducing postoperative nausea and vomiting and lowering overall medical costs.

****Potential risks**** that participants may face during this clinical trial include the possibility of still requiring supplemental analgesic or antiemetic medications.

6. ****Observation, Recording, and Management of Adverse Events****

If any adverse events occur during hospitalization, contact the physician for management. The investigator will follow up in a timely manner and update the database information accordingly.

7. **Research Quality Control and Assurance**

Ensure that the trial adheres to the clinical trial protocol and regulatory requirements, safeguards the rights and interests of the participants, and guarantees that trial records and reported data are accurate, complete, and credible.

8. ****Ethical Requirements****

The study must adhere to the Declaration of Helsinki and be conducted in accordance with Chinese regulations and norms governing medical research. Prior to the initiation of the study, the trial protocol must receive approval from the Ethics Committee of the lead medical research institution. Before enrolling any patient in the study, the investigating physician is responsible for providing a complete and comprehensive explanation, in writing, regarding the study's objectives, procedures, and potential risks to the patient or their designated representative. Patients must be informed of their right to withdraw from the study at any time. Each patient must be provided with a written informed consent form prior to enrollment. The investigating physician is responsible for obtaining informed consent from each patient before their participation in the study. The signed informed consent forms shall be retained as essential trial documentation for future reference.

**9. Research Team Members**

| Serial Number | Name | Affiliation | GCP Training Completed | Research Role |
| --- | --- | --- | --- | --- |
| 01 | Jiaojiao  Deng | Shihezi University School of Medicine | No | Case collection, data processing, manuscript writing |
| 02 | Hong  Zhang | The First Affiliated Hospital of Shihezi University | Yes | Research guidance |
